# Does Fecal Microbiota Transplant Have a Role in Treating Recurrent *Clostridioides difficile* Infection in Rural Hospitals?

**DOI:** 10.3389/fpubh.2021.670941

**Published:** 2021-06-09

**Authors:** Krishna Vedala, Philip Sobash, Parth Shah, Gilbert-Roy Kamoga

**Affiliations:** Department of Internal Medicine, White River Health System, Batesville, AR, United States

**Keywords:** *Clostridium difficile* infection, rural healthcare, health economics, fecal microbiota transplant, recurrent C difficile infection

## Abstract

*Clostridioides difficile* infection possesses a significant economical burden, specifically in the inpatient and rural settings. Fecal Microbiota Transplant has been used for treatment of recurrent *Clostridioides difficile* but its utility is limited by current guidelines and resources. We conducted a retrospective chart review to evaluate the financial benefit of using Fecal Microbiota Transplant after first recurrence of *Clostridioides difficile* infection. We found that while its use was restricted, on average Fecal Microbiota Transplant can save $11,603.49 per patient. In conclusion, our study shows that using Fecal Microbiota Transplant could prove to be economically beneficial in treating recurrent CDI in rural hospitals.

*Clostridioides difficile* infection (CDI) is one of most common nosocomial infections (1). *Clostridioides difficile* is a gram-positive, spore-forming anaerobic bacteria (2). It is often found in mammalian intestinal tract and also in the environment and is transmitted by fecal-oral route (2). Symptoms can range from being asymptomatic to varying degrees of diarrhea to even toxic megacolon and fatal colitis (2). While global estimated costs have been difficult to assess, it is estimated that in 2012 CDI placed nearly a $6.3 billion burden in the United States alone (3). The major risk factors of CDI are hospitalization, older age, and antibiotic exposure (2). Treatment of CDI is initially composed of oral antibiotics, specifically vancomycin (4). In the past, metronidazole was the first-line drug in non-severe CDI, but studies since then have shown that vancomycin is superior to metronidazole (2). Fidaxomicin, which was released into the market in 2011, has also been shown to be effective against CDI (2). Gut microbiota plays a significant role in various chemical processes while antibiotics alter the microbiota composition leading to mucosal immune defects and further enteric infections such as *Clostridioides difficile* (2). Fecal Microbiota Transplantation (FMT) is the placement of processed stool from a healthy donor into the intestinal tract of a patient with recurrent CDI (5). Short-term side effects of FMT can include gastro-intestinal symptoms such as nausea, vomiting, or diarrhea, transmission of enteric pathogens and complications of endoscopy (6). Long-term complications of FMT can include post-infectious irritable bowel syndrome, transmission of unrecognized infectious agents and induction of chronic disease based on altering gut microbiota (6). However, despite these side effects, FMT is often viewed as a very safe therapeutic technique (6). Approved by the FDA in 2013, FMT has shown to be effective in treating CDI (6). Current guidelines state FMT is recommended with the 3rd or subsequent non-severe recurrence of CDI (7). Since the approval of Patient Protection and Affordable Care Act (PPACA), hospitals especially in rural areas have been forced to deal with the economic ramifications of rising healthcare costs (8). Here, we discuss the results of a chart review to assess the effectiveness of FMT for treatment of CDI after first recurrence in terms of reducing healthcare costs in a rural setting.

We conducted a retrospective chart study utilizing assistance from our institution's IT department. We obtained data on all patients admitted with any CDI between the years of March 2015 and March 2020. In addition, we also requested data on all patients between that specific time period who underwent fecal microbiota transplant. With assistance from the billing department, data was abstracted utilizing the following ICD10 codes A04.71and A04.72 (for enterocolitis due to *Clostridioides difficile* recurrent and not specified as recurrent), CPT code 44705 (for preparation of fecal microbiota instillation) and HCPCS code G0455 (preparation with instillation of fecal microbiota). Data was reviewed and assessed using Microsoft Excel. Due to the study design, we did not have a need for any statistical analyses.

Our data shows that there have been 260 admissions for CDI over the past 5 years. We have excluded 140 admissions as they were not for recurrent admissions. A total of 80 admissions were for re-admission for CDI, with an average charge of $16961.92 for each of these admissions. Of these 80 admissions, ~43 cases were for more than one re-admission, qualifying for our proposed criteria for FMT after the first recurrence. Extended inpatient hospitalization contributed to the majority of these costs. Average length of hospitalization for each patient was ~3.5 days. However, our data showed that FMT has surprisingly been utilized only three times during the past 5 years averaging at a charge of $5358.43. Total expenditure of FMT included costs of endoscopic procedure (colonoscopy or sigmoidoscopy) and ancillary charges. All three patients who underwent FMT were discharged from hospital within the day of the procedure itself. Based on these calculations, we have shown that on average FMT can help save $11,603.49 (Table 1).

**Table 1:** Costs and savings associated with FMT and recurrent CDI.

	**Count**	**Average cost**
>1 Admission for recurrent CDI	43	$16,961.92
Fecal Microbiota transplant	3	$5,358.43
Potential savings		$11,603.49

*FMT, Fecal Microbiota Transplant; CDI, Clostridioides difficile infection*.

This study shows that despite the resources and significant prevalence of recurrent CDI in our community, FMT has not been utilized to its potential at our hospital. In addition, FMT overall appears to be safe with mild to moderate adverse events (9). FMT has also been shown to have a cure rate ranging from 70 to 90 percent (9). A recent prospective study showed that treatment with FMT can reduce risk of bloodstream infection, fewer days of hospitalization and increase in overall survival compared to those on antibiotic therapy (10). A 2018 study in Denmark even showed that total hospital costs dropped by 42% the first year after implementation of treating CDI with FMT (11). A major issue that often goes unstated is the plight of rural hospitals in the United States. Recent changes in the healthcare industry have placed small community and rural hospitals at risk of closure (8). Since 2011, ~74 hospitals in rural areas (in counties with no towns >10,000 people) have been closed (12). Although our findings are quite revelatory, this study does have its own limitations because we only took account of inpatient charges and have not incorporated other costs such as ambulatory prescription charges for antibiotics. Fidaxomicin is not available at our hospital, therefore all patients were treated with either metronidazole or vancomycin. This study was limited by current amount of FMT utilized at our hospital (data has revealed FMT only being utilized three times over past 5 years). Our analysis does not take into account all relevant health outcomes and treatment variations. In the near future, our plans include conducting further studies for more data for calculating true cost benefit of FMT, calculating QALY's and DALY's, and also applying our study design and collaborating with other local rural hospitals.

## Data Availability Statement

The datasets presented in this article are not readily available because our Institutional IRB approval does not allow sharing of confidential patient data to external institutions or individuals. Requests to access the datasets should be directed to kvedala@wrmc.com.

## Author Contributions

KV, PSo, and PSh collected data, interpreted data, and write the manuscript. G-RK helped oversee the entire project and proofread the manuscript. All authors contributed to the article and approved the submitted version.

## Conflict of Interest

The authors declare that the research was conducted in the absence of any commercial or financial relationships that could be construed as a potential conflict of interest.
